# Multi-receptor models for source identification of potentially toxic elements near antimony mines and assessment of their health risks

**DOI:** 10.3389/ftox.2026.1878246

**Published:** 2026-10-27

**Authors:** Danilo Čakmak, Veljko Perović, Dragan Čakmak, Dragana Pavlović, Marija Matić, Snežana Belanović Simić, Pavle Pavlović

**Affiliations:** 1 Faculty of Forestry, University of Belgrade, Belgrade, Serbia; 2 Department of Ecology, Institute for Biological Research “Siniša Stanković” - National Institute of the Republic of Serbia, University of Belgrade, Belgrade, Serbia

**Keywords:** antimony mines, health risk, potentially toxic elements, receptor models, Serbia

## Abstract

Soil in the area of the former antimony mine near the village of Lisa (Ivanjica municipality) was analysed in this study. The contents of potentially toxic elements (PTEs), as well as their relation to the natural background and the prescribed limit values, indicate that As exceeded the limit values in 63% of the samples, while in 37% of the samples it exceeded the remediation values. Using multivariate methods, including Network analysis, compositional data analysis, and positive matrix factorisation, three main sources of PTEs were identified. Arsenic was identified as the dominant pollutant, primarily associated with anthropogenic sources, but also partly with pedological factors. The health risk assessment showed that As is the main contributor to the overall risk, particularly for children, for whom the average health index (HI) is 2.89, almost three times higher than the permissible value. Regarding carcinogenic risk, As was also identified as a key factor, with an average value of 6.10E-05, indicating a notable risk. The highest contribution to HI comes from the anthropogenic factor, both for non-carcinogenic risk in children and for carcinogenic risk, while no significant risk was observed for the geogenic factor.

## 1 Introduction

Soil, as a key component of ecosystems, is increasingly under pressure due to industrialisation. One of the most important pollutants affecting soil health (SH) is potentially toxic elements (PTEs) (Saljnikov et al., 2019; Pavlović et al., 2021; Belanović Simić et al., 2023; Matić et al., 2023; Perović et al., 2025). These elements persist in the environment for long periods, as they are not degradable and exhibit low mobility in soil due to strong binding to soil particles (Sastre et al., 2002; Cakmak et al., 2018; Pavlović et al., 2018). The main natural source of PTEs is the geological substrate; however, it does not generally represent a serious environmental problem on its own, as ecosystems are naturally adapted to the presence of certain concentrations of these elements (Čakmak et al., 2020). In contrast, anthropogenic sources, although occupying smaller areas, can significantly increase PTE concentrations in soil and thereby disturb ecological balance. This is particularly important for the human population, especially in industrial areas where population density is higher (Sieghart et al., 2005; Islam et al., 2015).

Mines are significant sources of environmental pollution, with antimony mines being particularly notable (Cakmak et al., 2014; Belanović Simić et al., 2023). Their specificity lies in antimony deposits, which are often associated with other PTEs, which can be released into the atmosphere or accumulate in tailings during ore exploitation and processing (Bech et al., 2012; Čakmak et al., 2025a). In these economic ore deposits, Sb constitutes the primary ore mineral, with hazardous elements such as Cd, Pb, and especially As frequently occurring as paragenetic or associated elements within the Sb mineralisation matrix (Bech et al., 2012; Belanović Simić et al., 2023). One such site is the Lisa mine, near the town of Ivanjica in southwestern Serbia. The mine was closed in the mid-20th century, with the most intensive period of exploitation during the 1930s (IM, 2026). The impact of this mine on the surrounding soil has previously been documented (Cakmak et al., 2014).

For a more comprehensive understanding of the distribution of PTEs in soil and the assessment of the impact of mining activities on their content, various receptor models and statistical methods are applied. Network analysis (NA) is a centroid analytical method that uses a network structure to represent the relationships between elements and their connectivity, providing insight into the impact of pollutants on soil processes (Matić et al., 2023; Perović et al., 2025). It was originally applied in sociological studies (Adah et al., 2013), but due to its flexibility and ability to analyse relationships between different datasets and the dynamics of these interactions, it has since found wide application in the biological sciences (Hewavitharana et al., 2019), in studies of soil quality and land use management (Martín-Sanz et al., 2022; Zahedifar, 2023), as well as an effective tool for analysing interactions between PTEs in soil (Albuquerque et al., 2017; Matić et al., 2023; Čakmak et al., 2025a). Compositional Data Analysis (CoDA) is a receptor method suitable for identifying geochemical sources of individual elements, enabled by the application of the isometric log-ratio (ilr) transformation. It is particularly useful for studying the geochemical influences of individual elements in areas affected by mining activities (Čakmak et al., 2025a). The Positive Matrix Factorization (PMF) model is a receptor method first described by Paatero and Tapper (1994), which is also widely used for identifying sources of individual PTEs (Yuan et al., 2023). Although it is frequently applied in source apportionment of PTEs in soil (Vaccaro et al., 2007; Xue et al., 2014), its use is often complemented by other approaches to better capture complex inter-element relationships and environmental processes that cannot be resolved solely through factor decomposition (Čakmak et al., 2023).

In addition to the impact of mining activities, specifically microelements in soil, it is particularly important to assess their effects on the population living in the vicinity of the mine, considering that toxic particles can affect human health through three distinct exposure pathways: ingestion, dermal contact, and inhalation. For this purpose, the Health Risk Assessment (HRA) method is applied (USEPA, 1989; USDOE, 2011), Beyond assessing the overall risk of individual microelements, it is also important to analyse their combined effects (clusters) originating from mining activities, in order to evaluate the potential risk of chronic and carcinogenic diseases.

This study was designed to provide a comprehensive assessment of: (i) the degree of enrichment of individual PTEs in soil, (ii) the impact of mining activities on their occurrence and distribution, and (iii) their potential effects on human health.

## 2 Materials and methods

### 2.1 Study area and sampling

The study was conducted in the geographical area between 43°37′40″N and 43°39′08″N latitude and between 20°11′22″E and 20°13′47″E longitude, covering a total area of 8.6 km^2^. The study area comprises the village of Lisa in western Serbia, located in the immediate vicinity of Ivanjica. Samples were collected near a former antimony mine that operated until 1952 (IM, 2026). In total, 27 soil profiles were excavated in the study area. The sampling design was based on preliminary investigations for the preparation of a detailed soil map at a scale of 1:10,000, corresponding to a sampling density of approximately 3.14 profiles km^-2^. Samples for the determination of PTEs were taken from the upper A horizon. The location of each profile was determined using a GPS device (Figure 1).

**FIGURE 1:**
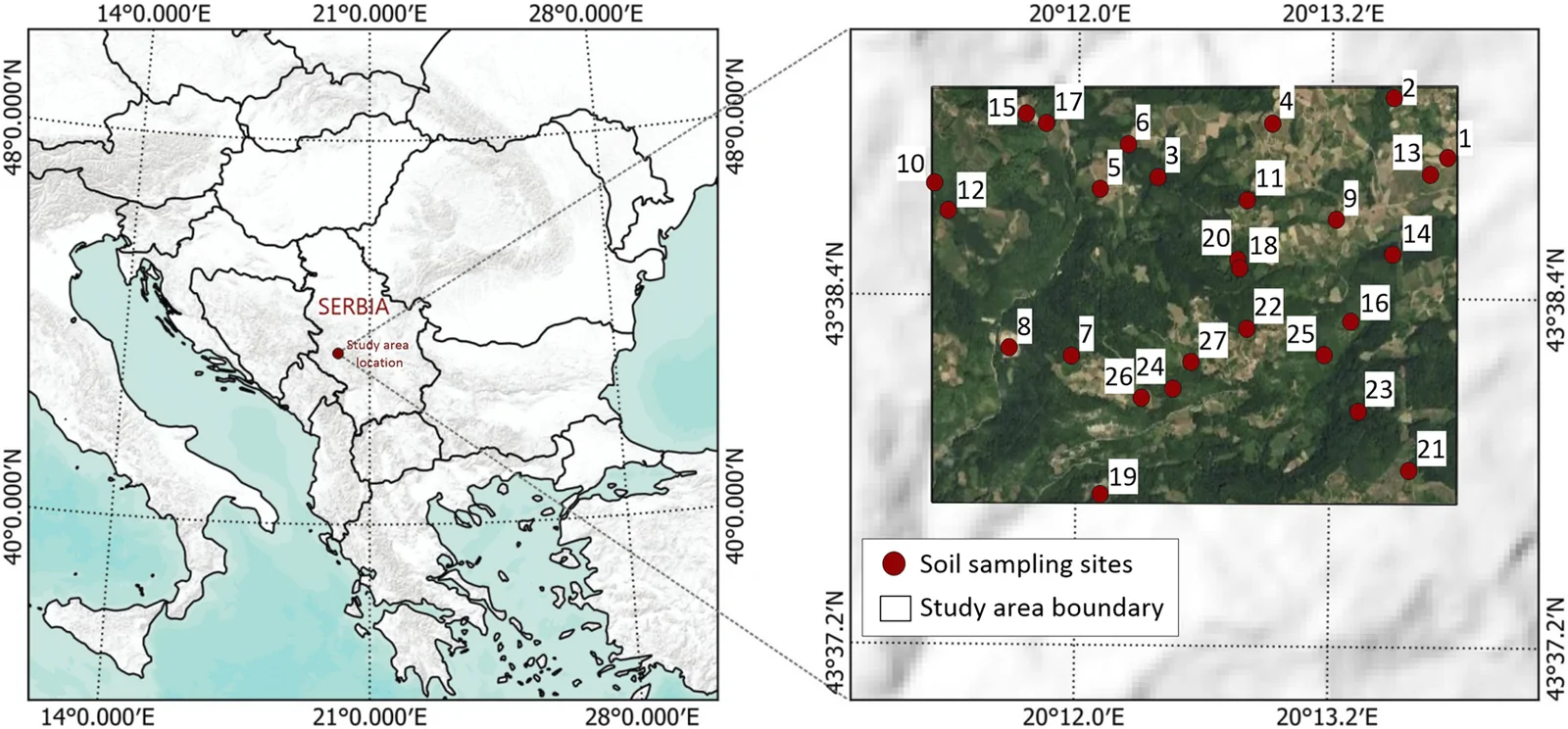
Study area and locations of the 27 soil sampling sites in the former antimony mining area near Lisa, western Serbia.

Field investigations indicated that the parent material consists of limestone, as well as chloritic and amphibolitic schists. After air-drying, the soil samples were ground in a mortar and sieved through a 2 mm mesh. Chemical analyses were then performed.

### 2.2 Soil analysis

The pipette method, according to the international B method, was used to determine particle size distribution (Gee and Or, 2002). Total carbon (C) was measured using a CHN analyser (Vario EL III, Elementar Analysensysteme GmbH, Hanau, Germany) according to the method of Nelson and Sommers (1996). After determining CaCO_3_ content with a Scheibler calcimeter, this value was subtracted from total C to obtain organic carbon. Hot acid-extractable PTEs were measured using an ICAP 6300 optical emission spectrometer (Thermo Electron Corporation, Cambridge, UK) after aqua regia digestion (ISO 11466:2004). The concentrations of As, Cd, Co., Cr, Cu, Fe, Mn, Ni, Pb, Zn, and Sb were determined. All chemical analyses were performed in triplicate. To verify the accuracy and reliability of the analytical results, a reference soil sample for microelements (ERM-CC141, clay soil, IRMM, Institute for Reference Materials and Measurements, Geel, Belgium) was analysed, with recoveries within 100% ± 15%. The limits of detection (mg kg^-1^) were: 0.6436 (As), 0.0166 (Cd), 0.0326 (Co.), 0.2897 (Cr), 2.3233 (Cu), 81.4905 (Fe), 0.4852 (Mn), 0.2239 (Ni), 2.8597 (Pb), 0.0851 (Sb), and 8.5106 (Zn). The relative standard deviation (RSD) was <5% for each element.

### 2.3 Assesment of degree of soil pollution

For the assessment of soil contamination, two key values defined in local legislation are used, based on the guidelines of the National Institute for Public Health and the Environment (SEDL, 2000), which address the protection of human health and the ecotoxicological effects of soil contaminants. The target value represents the level corresponding to the natural background of a given element (Supplementary Table S1), while the intervention value denotes the maximum permissible concentration, the exceedance of which requires the implementation of soil remediation measures. In addition to PTE concentrations, certain soil properties that influence element availability are also considered, such as organic matter content and particle size distribution (Equation 1).

$$\left(SW,IW\right)b=\frac{\left(SW,IW\right)*\lceil A+\left(B*clay\right)+\left(C*organic\;matter\right)\rceil }{\left[A+\left(B*25\right)+\left(C*10\right)\right]}$$ (1)

Where (SW)b represents target value (Supplementary Table S1) (IW)b represents intervention value (Supplementary Table S1), A, B and C are constant depending on the type of PTEs, as defined by European Union (EU) (SEDL, 2000) (Supplementary Table S1). Clay denotes the fraction of particles <2 μm, and organic matter (%) represents the organic matter content.

One of the measures of contamination is the individual ecological risk (Er), which indicates the degree of soil vulnerability caused by the presence of individual PTEs (Equation 2).

$$Er=Tr\frac{Cn}{GB}$$ (2)

where Tr represents the toxicity coefficient for a specific PTE, with values of As = 10; Cd = 30; Cr = 5; Cu = 5; Ni = 5; Pb = 5; Zn = 1 (Hakanson, 1980), and Sb = 7 (Wang et al., 2018); Cn represents the concentration of the element; and GB represents the background concentration of the element (MEP, 2016). The Er values are classified as follows: <40 low; 40–80 medium; 80–160 significant; 160–320 high; >320 very high (Hakanson, 1980).

The second indicator of contamination is the total ecological risk (RI), which is derived from the previously defined index and represents the sum of all individual ecological risks of the individual PTEs (Equation 3).

$$RI=\sum_{i=1}^{8}{Er}^{i}$$ (3)

The RI values are classified as follows: <150 low; 150–300 moderate; 300–600 considerable; >600 very high ecological risk (Hakanson, 1980).

### 2.4 Determining the sources of individual PTEs

Network analysis (NA) is a method based on linking independent variables, providing comprehensive insight into the relationships and dependencies between the studied elements. It consists of nodes, which represent the analysed variables, and edges, which represent their mutual relationships. Edges illustrate the connectivity between nodes and the strength of these relationships, visually expressed by the thickness of the lines. Green lines represent positive relationships, while red lines represent negative relationships (Robinaugh et al., 2016).

Compositional Data Analysis (CoDA) clr-biplot displays relationships between variables represented as points and vectors. This method is used for the analysis of geochemical variables (Liu et al., 2016; RGSCoDa, 2017; Thiombane et al., 2018). The length of the vectors represents the influence of individual variables, while their direction indicates the closeness between variables, i.e., their common origin (Thiombane et al., 2018). The clr-biplot was generated using CoDaPack software (RGSCoDa, 2017).

Positive Matrix Factorization (PMF) is a receptor method for identifying sources of individual PTEs, taking into account that part of their input in the study area originates from atmospheric transport and deposition. Unlike other receptor methods, this approach incorporates estimation of real error and does not allow negative values.

The model is represented by the following Equation 4:

X=GF+E (4)

Where X represents the nmx matrix (m measured chemical species in n samples), G represents the nxp matrix (source contributions in samples), and F represents the pxm matrix (source composition, i.e., source profiles). G and F represent factor matrices to be determined, while E represents the residual matrix, i.e., the difference between the X matrix and the model Y = GF as a function of G and F (EPA PMF v. 5.0). Method detection limits (MDLs) are used for the determination of measurement error (Equations 5, 6).

$$\mu ij=\sqrt{{\left(\sigma \;xconcentration\right)}^{2}+{\left(0.5xMDL\right)}^{2}}$$ (5)

If MDL≤ μij then:

$$\mu ij=\frac{5}{6}xMDL$$ (6)

where *uij* represents the uncertainty associated with the concentration of element *j* in sample *i*, MDL represents the method detection limit, and *σ* the relative standard deviation.

All 10 analyzed chemical species were classified as “Strong” variables. The Signal-to-Noise (S/N) ratios ranged from 5.76 (Cu) to 8.99 (Zn). No data points were excluded or downweighted, ensuring 100% data utilization. The optimal number of sources was determined by evaluating the Qtrue/Qexpected ratio across different factor solutions. The Qtrue/Qexpected value for a 2-factor model was high at 5.57, while for a 4-factor model it dropped to 2.18. Based on these mathematical diagnostics, alongside the distinct, physically meaningful chemical profiles, a 3-factor solution was selected as the optimal choice Qtrue/Qexpected approx 2.67. Base The PMF model achieved a 100% convergence rate across 20 independent base runs. The mathematical QRobust and QTrue values were highly stable (470.4 and 489.4, respectively). A minimal difference between them (<5%) indicates that the model was not distorted by extreme outliers. A Bootstrap (BS) analysis with 100 independent runs achieved a 100% convergence and a perfect 100% factor mapping rate for all three factors, confirming excellent statistical stability and reproducibility. Because the base configuration of the 3-factor system already demonstrated a 100% convergence rate and perfect bootstrap mapping, additional factor skewing via the Fpeak parameter was not required.

Maps of factor influence were also generated based on factor scores using ArcGIS software.

### 2.5 Determination of the health risk (HRA)

The impact of PTEs in soil on human health was assessed using the following equations: non-carcinogenic risk for ingestion (Equation 7), dermal contact (Equation 8), and inhalation (Equation 9).

$$HQing=\left[\left(CxIRSxRBAxEFxED\right)/\left(BWxATxRfDo\right)\right]x10^{-6}$$ (7)

$$HQder=\left[\left(CxSAxAFxABSdxEFxED\right)/\left(BWxATxRfDoxGIABS\right)\right]x10^{-6}$$ (8)

$$HQinh=\left[\left(CxEFxED\right)/\left(ATxRfCxPEF\right)\right]$$ (9)

Where total non-canceroginc risk is:

HI=HQing+HQder+HQinh (10)

Carcinogenic risk.

The carcinogenic risk for residents is calculated using the following Equations 11-15:

$$CRing=\left(\left(C\;x\;IFS\;x\;RBA\;x\;CSFo\right)|AT\right)\;x{\;10}^{-6}$$ (11)

$$IFS=\left(EF\;x\;EDa\;xIRSa|BWa\right)+\left(EF\;x\;EDc\;x\;IRSc│BWc\right)$$ (12)

$$CRder=\left(\left(C\;x\;DFS\;x\;ABSd\;x\;CSFo\right)|AT\;x\;GIABS\right)\;x{\;10}^{-6}$$ (13)

$$DFS=\left(EF\;x\;EDa\;x\;SAa|BWa\right)+\left(EF\;x\;EDc\;x\;SAc\;x\;AFc│BWc\right)$$ (14)

CRinh=C x EF x ED x IUR x 1000/AT x PEF (15)

The total carcinogenic risk (TCR) is the sum of the carcinogenic risks for each of the three exposure routes (ingestion, dermal contact and inhalation) is calculated using the following Equation 16:

TCR=CRing+CRder+CRinh (16)

The description and reference values for all parameters in the above equations are provided in Supplementary Tables S2 and S3 (USDOE, 2011), while the calculations of HQ, HI, and CR were performed using the USEPA RSL calculator (2020). For total carcinogenic risk (TCR), values below 10^−6^ indicate no risk, while values above 10^−4^ indicate unacceptable risk. TCR values in the range of 10^−6^ to 10^−4^ are considered a notable risk. For the health risk index (HI), values above one indicate unacceptable risk, while values below one indicate no risk. Otherwise, they represent a clear risk to human health.

The combination of the PMF and HRA models was developed to determine the contribution of each factor to the potential occurrence of chronic diseases and carcinogenic effects in the study area.

## 3 Results and discussion

### 3.1 Total content of examined elements and basic pollution indices

Considering the mean values of the elements, only two elements exceed the background values defined for the wider area of western Serbia, which includes the study area. These are As, which exceeds the background value by almost three times, and Cd. The mean As concentration recorded in the present study (44.53 mg kg^-1^) is generally comparable to those reported for soils surrounding other Sb mines, such as the Xikuangshan mine and an Sb mining area in Guangxi Province, China, where mean concentrations of 61 and 40.13 mg kg^-1^, respectively, were reported (Li et al., 2024; He et al., 2025). However, considerably higher As concentrations have also been reported in some Sb mining areas, such as Zuoxiguo, where the mean concentration reached 417.53 mg kg^-1^ (Zahedifar, 2023). At the level of individual samples, it is noticeable that As exceeds the guideline value in 63% of samples, while in 37% of samples it exceeds the remediation value (Table 1). The highest values were recorded in the southwestern parts of the study area, mainly in the hilly terrain (Supplementary Table S4; Figure 1). The occurrence of As an associated element of Sb, as well as its role as the main contaminant within the mine, has also been confirmed in previous studies (Belanović Simić et al., 2023; Čakmak et al., 2025a). Its very high standard deviation (Table 1) indicates an anthropogenic origin (Karim et al., 2014). For Cd, values above the limit values were recorded in 74% of samples, indicating that almost the entire study area is affected by elevated concentrations of this element (Supplementary Table S4). Based on the data obtained, it can be concluded that anthropogenic influence is present, especially considering the elements that occur as companions of Sb and are characteristically associated with mining activities in the surrounding area (Bech et al., 2012; Čakmak et al., 2025a). However, the standard deviation value lower than 50 (Table 1) indicates a significant contribution from geological origin. This influence is further supported by the limestone bedrock, which may be the cause of the elevated content of this element (Xia et al., 2020). For Sb, only 14.81% of samples exceeded the limit value, with elevated concentrations recorded in the central parts of the study area. However, standard deviation values slightly above 50 (Table 1) indicate the influence of anthropogenic factors (Karim et al., 2014). For the other elements, exceedance of the limit value was recorded only for Cr, in 3.70% of samples (one sample), while all other elements, including Cr, have standard deviation values below 50 (Table 1). These results are also reflected in the ecological risk for the whole area, where only 4%, i.e. 14.81% of samples showed values corresponding to moderate risk (Table 1).

**TABLE 1:** Descriptive statistics for potentially toxic elements (PTEs) and pollution indices.

Elements		As	Cd	Co	Cr	Cu	Ni	Pb	Sb	Zn
		mg kg^-1^								
Everage		44.53	1.68	12.63	73.50	18.22	34.42	27.09	2.91	64.00
Median		34.78	1.42	12.70	72.84	17.95	34.14	25.74	2.54	63.07
Max		135.85	3.39	18.83	144.84	44.32	66.46	52.50	8.59	97.00
Min		3.76	0.72	3.91	37.25	6.41	9.3	11.60	1.35	31.55
SD		39.51	0.73	3.52	25.32	7.75	14.82	10.40	1.46	16.42
CV %		88.76	43.10	27.86	34.45	42.52	43.04	38.39	50.13	25.65
Critical values		%*								
<Target value		37.04	25.93	100	96.30	96.32	100	100	85.19	100
> Target value		25.92	74.07	—	3.70	3.70	—	—	14.81	—
>Remediation value		37.04	—	—	—	—	—	—	—	—
Ecological Risk (Er)	Value	%*								
Low	<40	66.67	59.26	96.66	100	100	100	100	100	100
Medium	40–80	29.63	40.74	0.00	—	—	—	—	—	—
Significant	80–160	3.70	—	—	—	—	—	—	—	—
Total Ecological Risk (RI)	Value	%								
Low	<150	85.19								
Moderate	150–300					14.81				

In terms of ecological risk, the analysis of the investigated elements showed that most PTEs (Cr, Cu, Ni, Pb, Sb, and Zn) were associated with a low level of ecological risk. A similar pattern was observed for Co., with 96.6% of the samples classified as low risk. In contrast, As and Cd were identified as the most critical elements. Cd showed the highest proportion of samples in the moderate-risk category (40.74%), largely reflecting the influence of the geological substrate. As exhibited the widest range across risk categories. Although most samples (66.67%) were classified as low risk, 29.63% showed moderate risk and 3.70% reached the significant-risk category, indicating the occurrence of localized contamination hotspots. Overall, 14.81% of the samples were classified as having moderate total ecological risk, which may reflect the cumulative contribution of elevated Cd and As levels (Table 1).

The spatial distribution of total ecological risk further reveals a heterogeneous pattern (Figure 2), with the highest risk concentrated in the southwestern and central-western parts of the study area. These areas correspond to zones of elevated As and Cd concentrations. Transitional zones of elevated risk surrounding the main hotspots may reflect the redistribution of PTEs through local environmental processes, including water and wind transport and topographic effects. Overall, the spatial pattern indicates that elevated ecological risk is not uniformly distributed but is predominantly concentrated in the western and southwestern parts of the study area (Figure 2).

**FIGURE 2:**
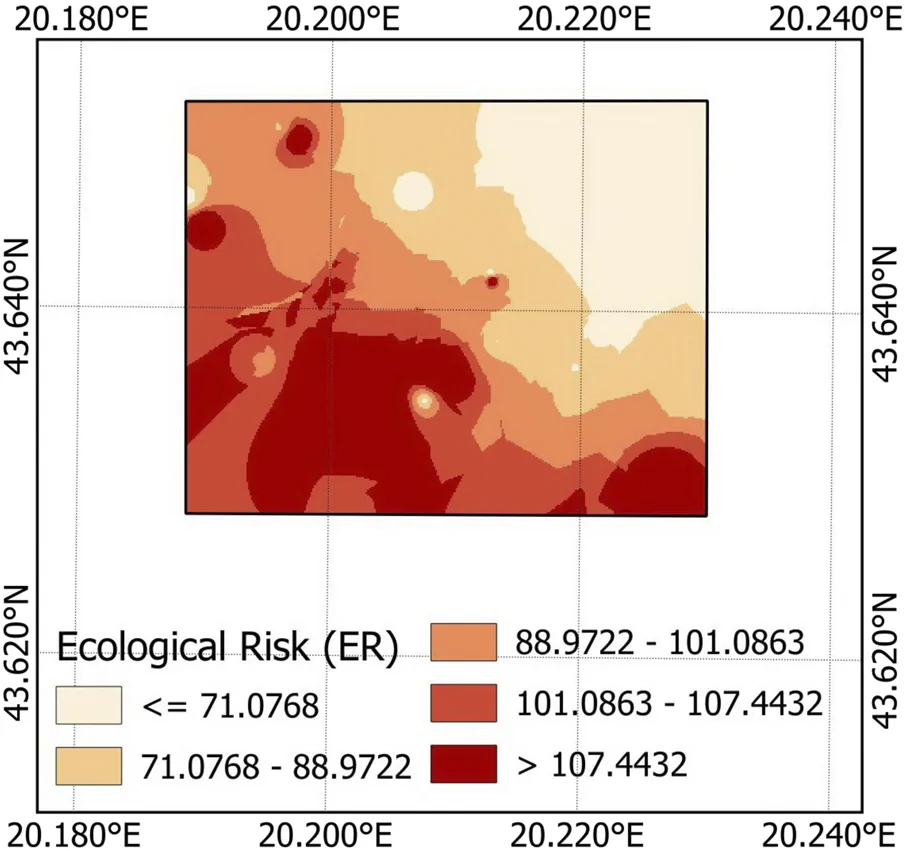
Spatial distribution of ecological risk (RI).

### 3.2 Determining the source of PTEs

#### 3.2.1 Network analysis (NA)

In network analysis, it is typical that, in addition to grouping elements by common properties and origin, it also provides insight into the types and strengths of relationships between individual groups. Unlike classic receptor models, which mainly focus on the common origin of elements, NA offers a more detailed representation of their mutual relationships. The analysis identified three clusters. The most prominent cluster is formed by Fe as the central element, which is most strongly connected to Cr and Cd, and more weakly to Zn. This cluster represents the soil component, given the presence of Fe, as well as Ni and Cr, which are characteristic of the entire area of western Serbia (MEP, 2016; Čakmak et al., 2018). The second cluster, dominated by Mn and Co., reflects the pedological characteristics of the soil, considering their affinity for mutual interactions in the processes of secondary mineral formation (Li et al., 2024). The third cluster consists of the partially isolated Sb and As, which show weak connections with the other groups and are most likely influenced by anthropogenic factors, in line with the specific characteristics of the study area (Čakmak et al., 2025a). The strength of the connections that Cd establishes with the group containing Fe and As, and to a lesser extent with Sb, clearly indicates its dual nature, with a more pronounced influence from the geological substrate (Xia et al., 2020).

The connections that Pb forms with As, Mn, and Cd indicate its anthropogenic origin, associated with ore extraction and processing (Bech et al., 2012; Čakmak et al., 2025a), but also its role in the formation of the soil cover, as well as its binding to Mn and soil organic matter as part of the geological substrate (Shuman, 1988; Hu et al., 2023). These elements, and especially Pb, act as bridges within the network, which is also reflected in higher values of betweenness centrality. Based on high values of expected influence, Fe and Mn stand out as the main components whose participation in redox processes affects the behaviour of other elements (Rotter et al., 2017). In addition, Cd also shows high values for this parameter, which can be explained by its pronounced activity in adsorption and desorption processes in soil (Kubier et al., 2019). Considering the highest connectivity of Pb, Cd, As, and to a lesser extent Fe, the values of closeness centrality indicate their more central position within the network and more efficient propagation of influence across elements. Their pronounced interconnection also indicates their impact on the largest number of elements in the network, as confirmed by the values of strength (Figure 3A; Figure 3B). In the case of As, in addition to indicating its anthropogenic origin, its strong connection with Pb also suggests its involvement in pedological processes, which can be associated with atmospheric deposition and the long period of mine exploitation (Pejović et al., 2017).

**FIGURE 3:**
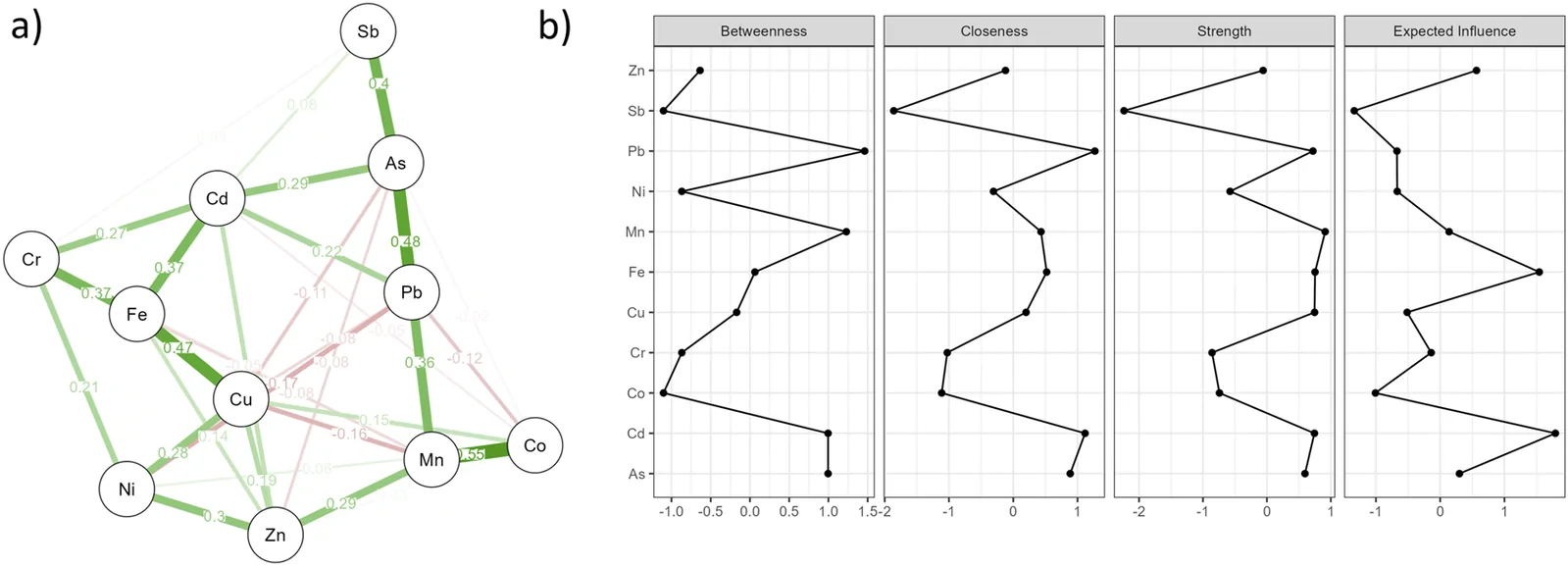
**(A)** Network analysis (NA) of potentially toxic elements (PTEs), where nodes represent individual elements and edges represent their relationships; **(B)** Network characteristics of individual PTEs.

#### 3.2.2 Compositional data analysis (CoDA)

The results of the CoDA analysis clearly indicate the separation of three clusters in a geochemical context. The first cluster, with the most pronounced influence, is formed by As, where the sharp angle with Sb indicates their common origin. Within this cluster, points 12, 17, 18 and 27 are particularly prominent (Figure 4). Such a role of As is common (Čakmak et al., 2025b), so it can be said that, according to the strength of influence (and not in the exploitation sense), Sb is actually a companion of As (Belanović Simić et al., 2023; Čakmak et al., 2025a). For As and Sb, it is characteristic that they occur in transitional contact zones between limestone and magmatic rocks, where geothermal sources often occur, enabling their mobilisation and release (Strmić Palinkaš et al., 2018). This results from their association with magmatism and extensional tectonic settings, as well as the presence of carbonate host rocks and characteristic geochemical and alteration signatures (Hofstra and Cline, 2000). The second cluster, consisting of Fe, Cr, Ni, Cu and Zn, reflects the characteristics of the geological substrate of this area, considering the dominance of ultramafic rocks and the presence of chlorite-siliceous and amphibole schists in western Serbia, which are typically enriched in Cr and contain moderate concentrations of Ni (Banakeng et al., 2016). The points that stand out under the influence of this cluster are 9, 13 and 14. The third cluster is displaced relative to the first, indicating a different geochemical origin. Additionally, the vertical position of the Mn vector (Figure 4) clearly indicates its different origin compared to the other analysed elements. However, Pb, positioned between As and Mn, forms a link between these groups, with points 5 and 6 identified in that area. As previously mentioned, Mn displays independent behaviour, with points 15, 16, and 20 typically influenced by it. Cobalt is also observed, with points 1 and 4 strongly influenced by this vector, while its position relative to the vector comprising Fe, Cu, Cr, and Ni suggests a partially common origin (Figure 4).

**FIGURE 4:**
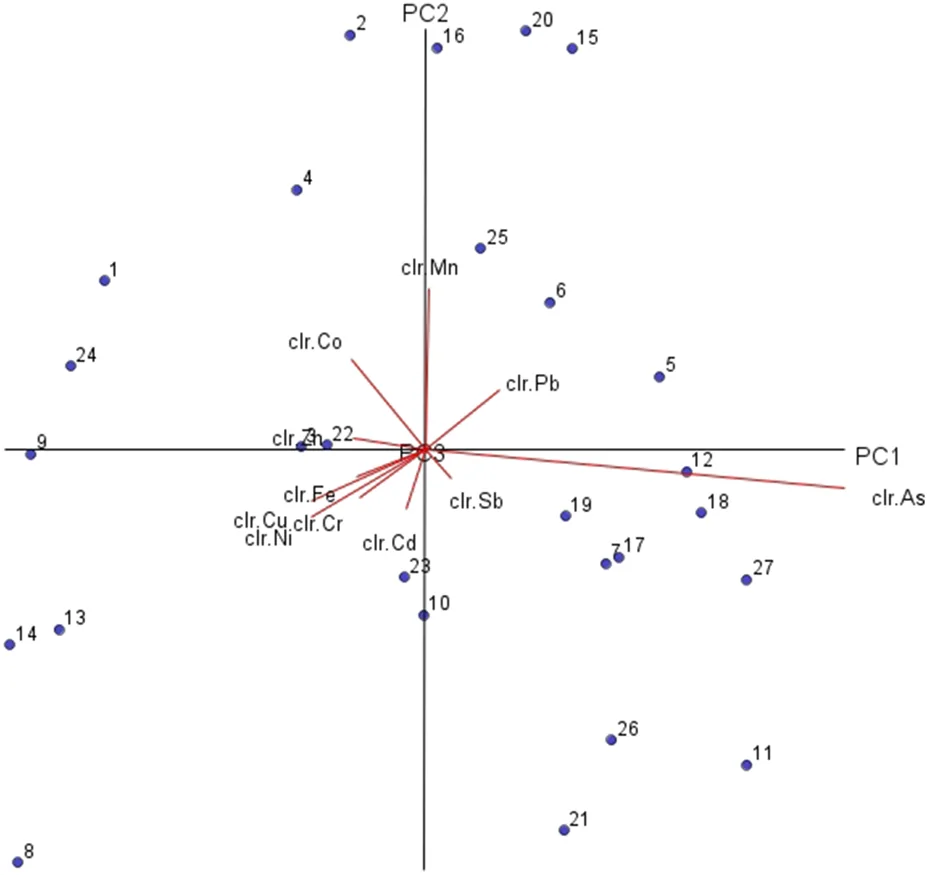
k-means clustering of potentially toxic elements (PTEs) based on the compositional data analysis (CoDA) centered log-ratio (clr) biplot.

#### 3.2.3 Positive matrix factorization PMF

Of the three factors identified by the PMF model, the third factor shows a significantly higher contribution, approximately twice as large, to the total variance compared with the first and second factors, whose contributions are nearly equal. This relationship can be explained by the fact that the PMF model is based on mass contributions, with Fe being predominantly associated with the third factor, leading to this disparity and representing one of the limitations of the PMF approach. Factor I is characterized by dominant contributions of Mn, Co., and Pb, accounting for 77.5%, 59.2%, and 50.8%, respectively. This factor can be more precisely defined as a pedogenic factor controlled by sorption processes. Secondary Mn oxides and hydroxides in soils have a high affinity for binding trace metals. Therefore, the substantial contribution of Pb and, to a lesser extent, As to this factor does not necessarily indicate direct anthropogenic inputs but may reflect their adsorption and accumulation by Mn oxides during pedogenesis (Li et al., 2024; Pejović et al., 2017), clearly distinguishing this factor from the primary geological influence.

The interpretation of Factor I is further supported by the other receptor models. In the network analysis (NA), the Mn–Co relationship is represented by the strongest positive connection in the network, while the Mn–Pb association further supports the role of Mn in sorption processes. Similarly, in the CoDA/PCA space, the Mn and Co. vectors show a similar orientation along PC2 and are clearly separated from PC3 and, particularly, PC1. The consistency among PMF, NA, and CoDA therefore supports the interpretation of Factor I as a Mn/Co-rich pedogenic factor dominated by secondary sorption processes.

The second factor is characterised by a distinctly high loading of As (>80%), along with significant contributions from Pb (40%) and Sb (37%), with Sb also being the most represented in this factor. Such an association of elements is characteristic of anthropogenic influence in the vicinity of an antimony mine (Bech et al., 2012; Peng et al., 2026).

The NA results are fully consistent with the PMF model. In the network diagram, Sb and As are strongly associated within a distinct chain that is further connected to Pb. The strong positive relationships among these elements indicate their common spatial and geochemical origin. This pattern is also supported by the CoDA/PCA model, where Sb, As, and Pb are oriented towards PC2, with a particularly strong contribution of As, indicating a common pattern within the dataset associated with mining activities.

In the third factor, the highest contributions are observed for Ni (67%), Cu (65%), Cr (54%), and Fe (54%). The pronounced influence of Fe, Cr, and Ni clearly indicates the pedological substrate, that is, the influence of magmatic rocks (Banakeng et al., 2016). Within this factor, a slightly higher contribution of As is also observed, which, as in the first factor, can be attributed to its input into the soil as a result of mining activities and ore processing, as well as its further integration into pedogenetic processes. A significant share of Sb (35%) is a consequence of its geological origin, considering that its deposits in this area occur at relatively shallow depths (IM, 2026; Table 2).

**TABLE 2:** Source composition of PTEs (%) from the PMF model.

Species	Factor I	Factor II	Factor III
As	19.17	80.83	0.00
Cd	19.75	33.23	47.02
Co.	59.16	15.67	25.18
Cr	19.46	26.71	53.84
Cu	15.44	19.72	64.84
Fe	23.87	24.73	51.40
Mn	77.51	22.49	0.00
Ni	11.30	21.71	67.00
Pb	50.78	40.40	8.83
Sb	27.64	37.17	35.19
Zn	33.80	20.52	45.68
Expleined variance (%)	25.20	24.77	50.02

The NA also clearly distinguishes a cluster comprising Ni, Cu, Cr, Fe, Zn, and Cd, supporting their grouping as elements predominantly associated with a geogenic source and, more specifically, with the serpentinitic parent material. The CoDA/PCA biplot further supports this geochemical association, as these elements are grouped around the PC3 axis, indicating a common lithogenic control.

Overall, the consistent results obtained from NA, CoDA, and PMF demonstrate that their integration enables a clearer differentiation of the main geochemical processes and PTE sources in the study area.

In terms of spatial distribution, Factor I exhibits a nearly uniform presence across the entire study area, confirming its pedological character. Factor II is dominant in the north-eastern parts, while Factor III is prominent in the central zone, oriented in a north–south direction (Figure 5).

**FIGURE 5:**
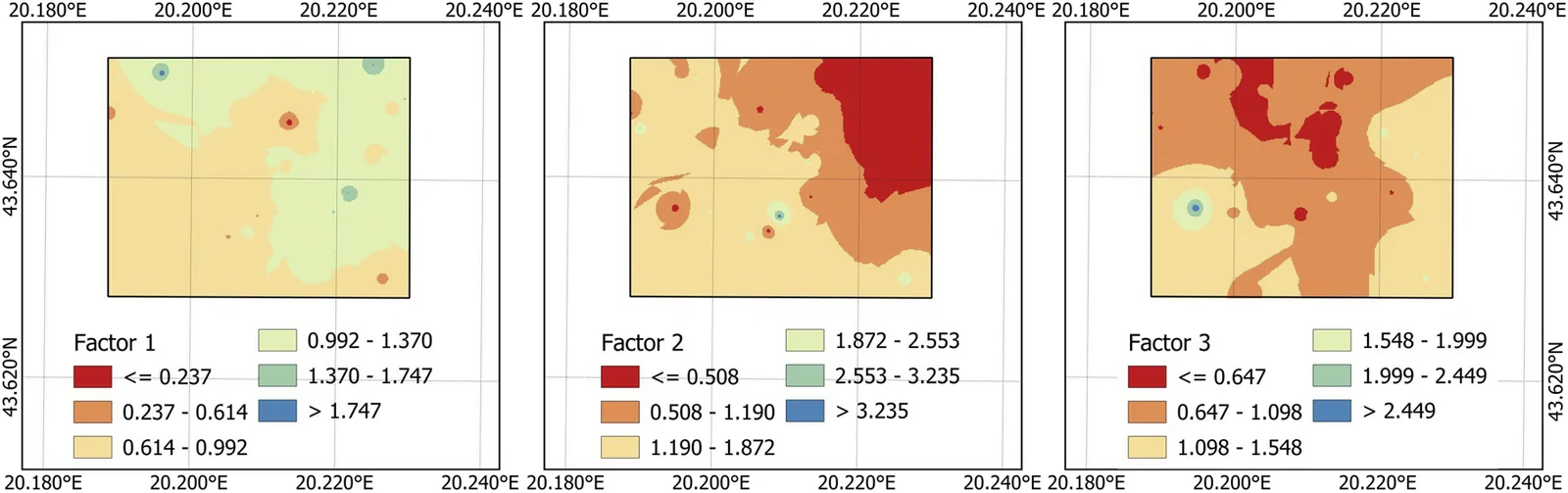
Spatial distribution of factor scores for the three sources identified by positive matrix factorization (PMF): Factor I (pedogenic), Factor II (anthropogenic/mining-related), and Factor III (lithogenic/litho-pedogenic).

#### 3.2.4 Source oriented health risk assessment

The average total HI value is 2.89 E+00, nearly three times higher than the threshold considered acceptable (Figure 6). The maximum value of 5.95E+00 is almost six times the upper acceptable limit (Supplementary Table S5). For individual elements, only As, with an average value of 1.28 E+00, indicates a potential risk of chronic diseases, with approximately 50% of samples exceeding the value of 1 (Supplementary Table S5). This result is expected for areas near Sb mines, given the elevated As content in their surroundings (Čakmak et al., 2025a). In the previously mentioned Sb mining areas, the HI value for children in the vicinity of the mine in Guangxi Province was 5.82E+00, which is considerably higher (He et al., 2025), and particularly in the vicinity of the Zuoxiguo mine, where a value of 1.95E+01 was reported (Bai et al., 2023).

**FIGURE 6:**
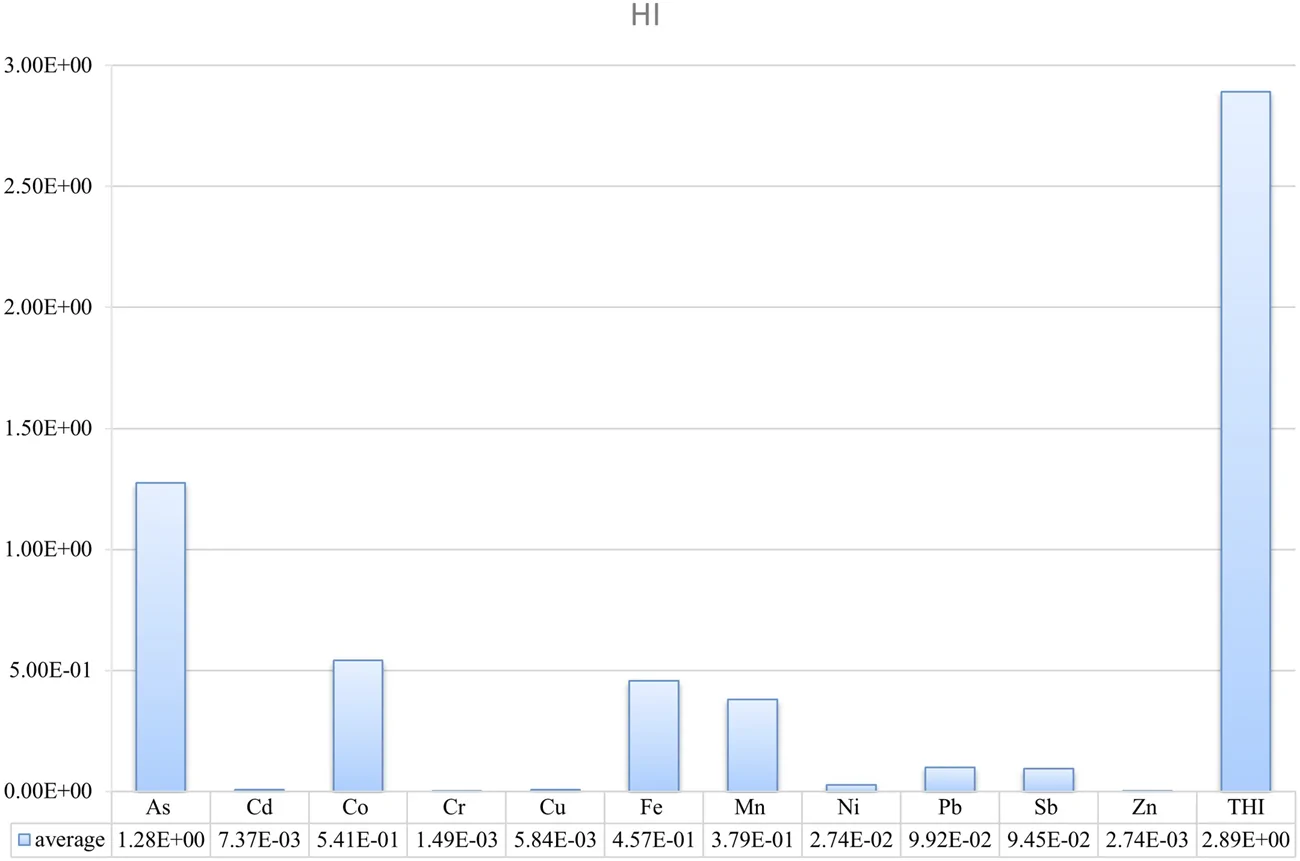
Average non-carcinogenic risk for children (HI).

The average total HI value for adults is 3.07E−1, indicating that the adult population is not exposed to a risk of chronic effects in the studied area. Furthermore, the maximum value does not exceed the reference risk threshold (Figure 7). In the study by He et al. (2025), no potential risk of chronic effects was identified for adults, whereas Bai et al. (2023) reported values above the safety threshold, reaching 2.15E+00.

**FIGURE 7:**
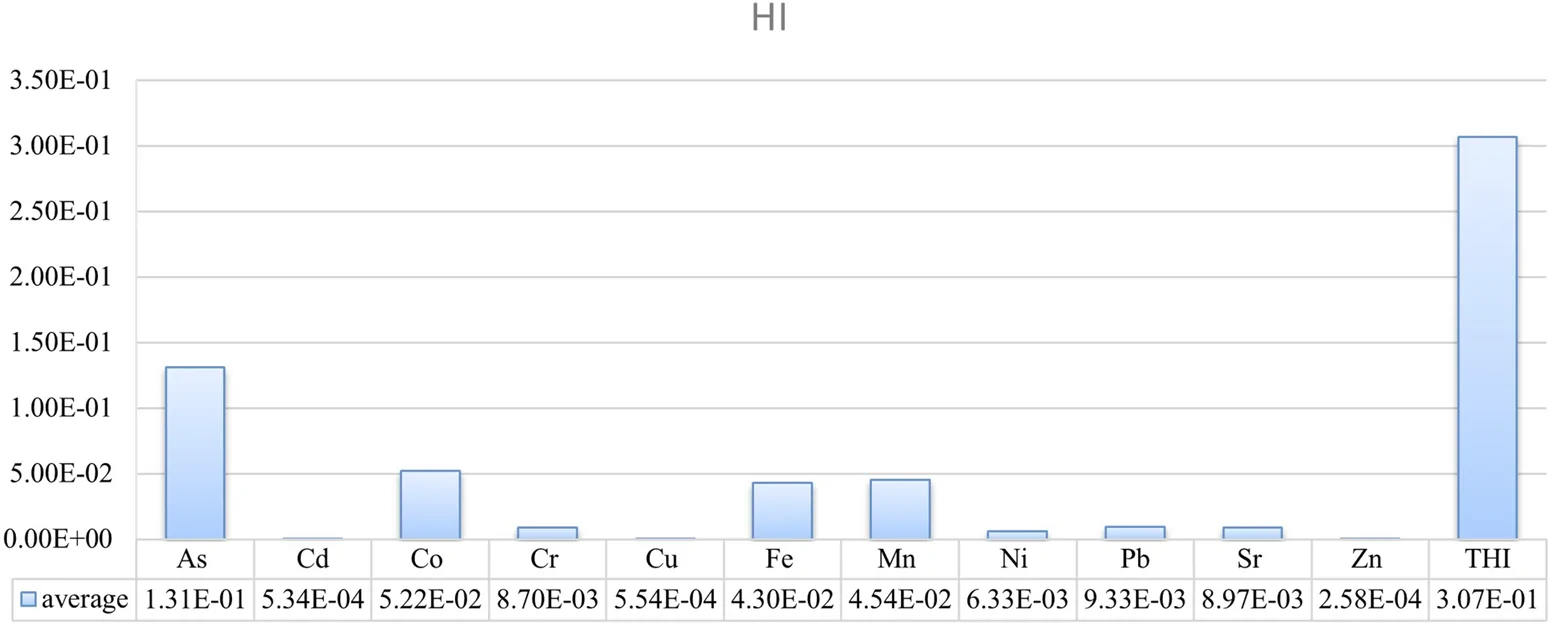
Average non-carcinogenic risk for adults (HI).

The average carcinogenic risk falls within the notable risk range and amounts to 6.10E−05, with the maximum value also in this category. As with the non-carcinogenic risk in children, As is the main contributor, with its average value also within the notable risk range, and the maximum value remaining in the same range (Supplementary Table S6). Of all samples, 74% fall within the notable risk category (Figure 8; Supplementary Table S8). Regarding As, He et al. (2025) also reported a notable carcinogenic risk, with a value of 9.99E-05, whereas Bai et al. (2023) reported an unacceptable risk, with a value of 3.39E-4.

**FIGURE 8:**
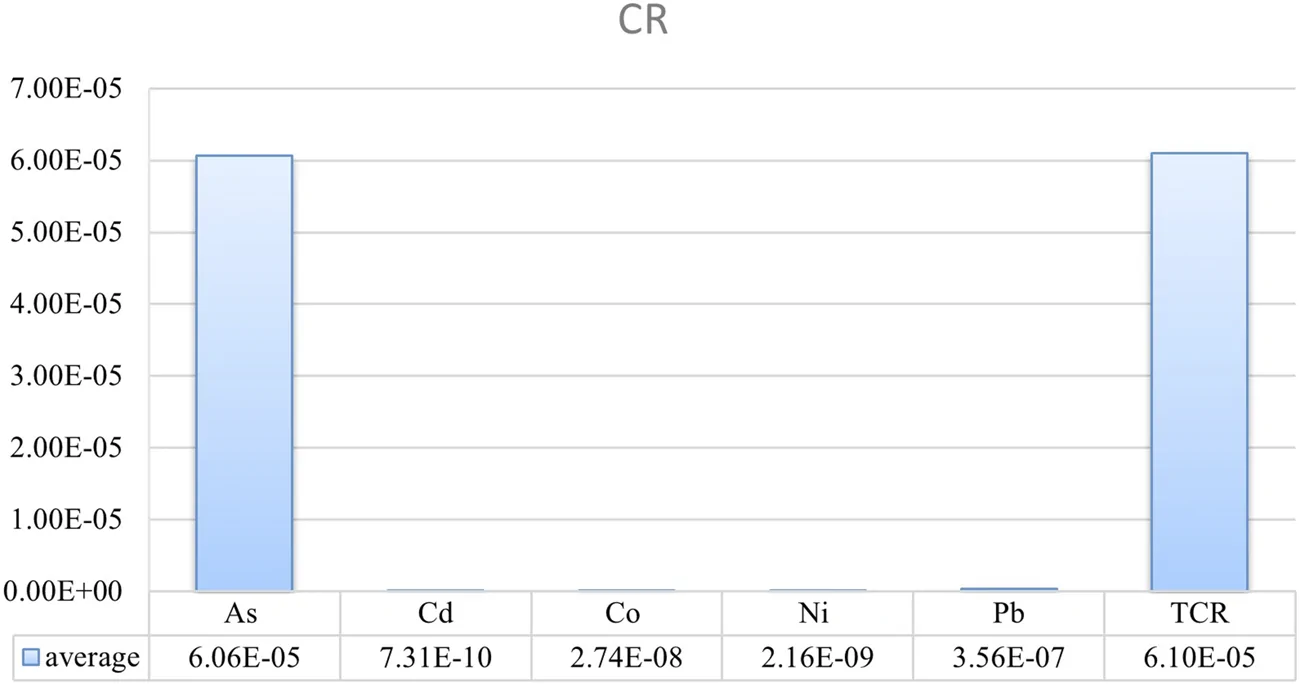
Average cancer risk (CR).

According to the PMF model, three factors were identified. In the assessment of non-carcinogenic risk for children, the highest value was recorded for Factor II at 1.40 E+00, while the maximum individual value reached 3.65 E+00 (Supplementary Table S7). For Factors I and II, 44.44% of individual values do not exceed the threshold value of 1.00 E+00. For Factor III, none of the values exceed 1.00 E+00, with a maximum of 6.68E-01 and an average of 4.41E-01 (Figure 9A; Supplementary Table S7). Regarding the contributions of individual elements, As dominates in Factors I and II, with mean values of 1.03 E+00 and 2.45E-01, respectively. In Factor II, compared to Factor I, Co., Mn, Pb, and Zn are also identified as significant contributors (Supplementary Figure S1).

**FIGURE 9:**
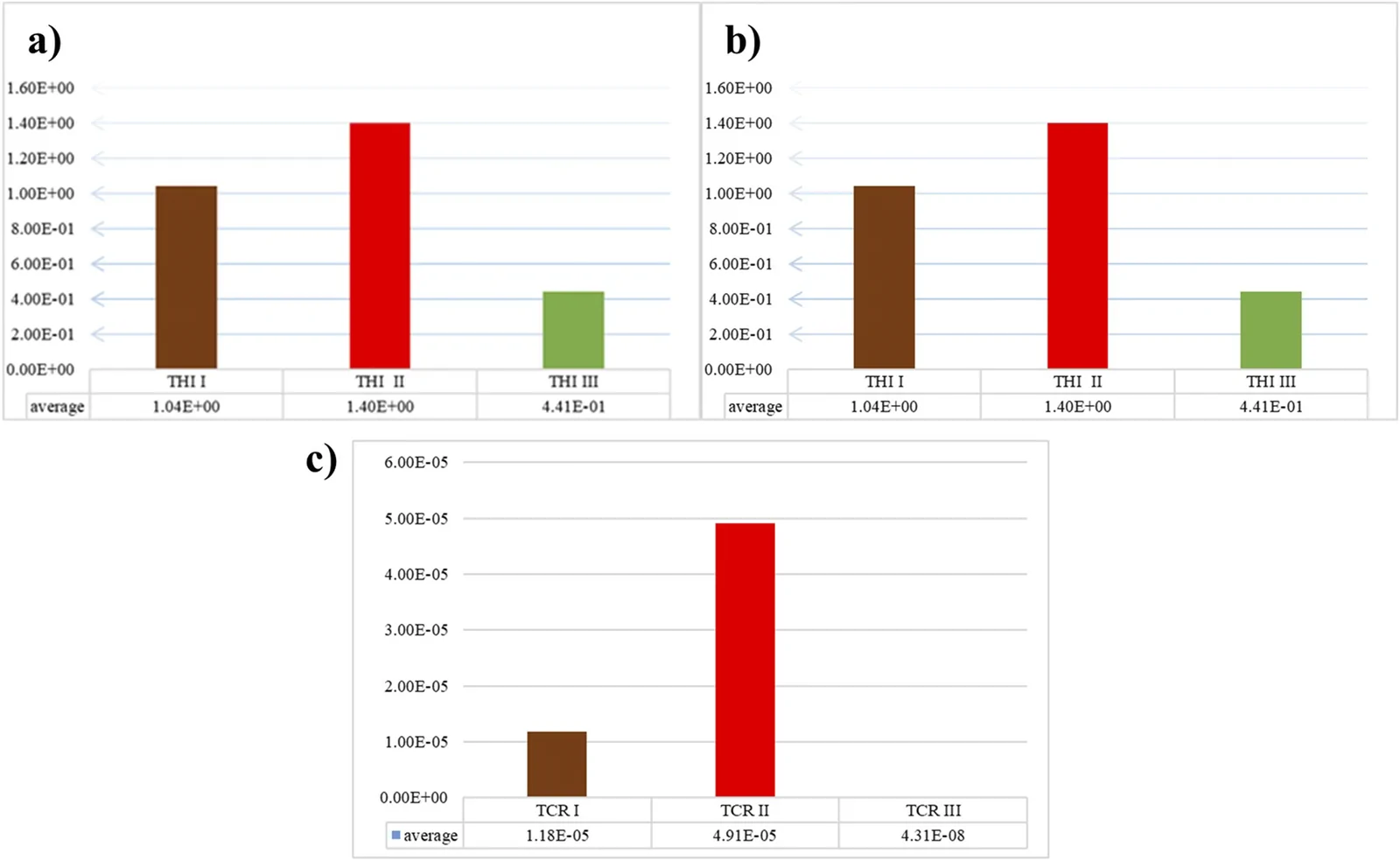
Health risk values by factors: **(A)** non-carcinogenic risk for children; **(B)** non-carcinogenic risk for adults; **(C)** carcinogenic risk.

In the assessment of non-carcinogenic risk for adults, the mean THI values for Factors I and II exceed the reference threshold of 1.00 E+00, while for Factor III this value remains below the limit. The highest mean value was recorded for Factor II (THI = 1.40 E+00), followed by Factor I (THI = 1.04 E+00), and the lowest value was determined for Factor III (THI = 4.41E-01) (Figure 9B).

In the assessment of carcinogenic risk, the values for Factors I and II fall within the notable risk range, while for Factor III the risk remains within safe limits. The highest mean value was recorded for Factor II (4.91E−05), followed by Factor I (1.18E−05), and the lowest for Factor III (4.31E−08), with even the maximum values for this factor not indicating any risk (Figure 9C). Considering individual values, neither Factor I nor Factor II exceeds the threshold of unacceptable risk. However, for Factor II, a high proportion of samples exceed the notable risk threshold (74.07%), while for Factor I this proportion is lower (48.15%). The largest contribution to the total carcinogenic risk is from As, with significantly higher values compared to other elements and a dominant contribution within Factors I and II. This is followed by Pb and Co., and then Ni and Cd (Supplementary Table S7; Supplementary Figure S2). It is also important to note that As speciation, distinguishing between inorganic and organic forms, was not performed in this study, which represents a common limitation of preliminary environmental risk assessments. The application of the USEPA RSL approach assumes a worst-case scenario based on inorganic As. This conservative approach enables the identification of potentially critical areas that should be prioritized for further investigation.

Regarding the spatial distribution of total HI, the highest intensity is observed in the northeastern part of the study area. Although all factors show elevated values in this area, Factor II stands out terms of influence, indicating its dominant spatial contribution (Figure 10).

**FIGURE 10:**
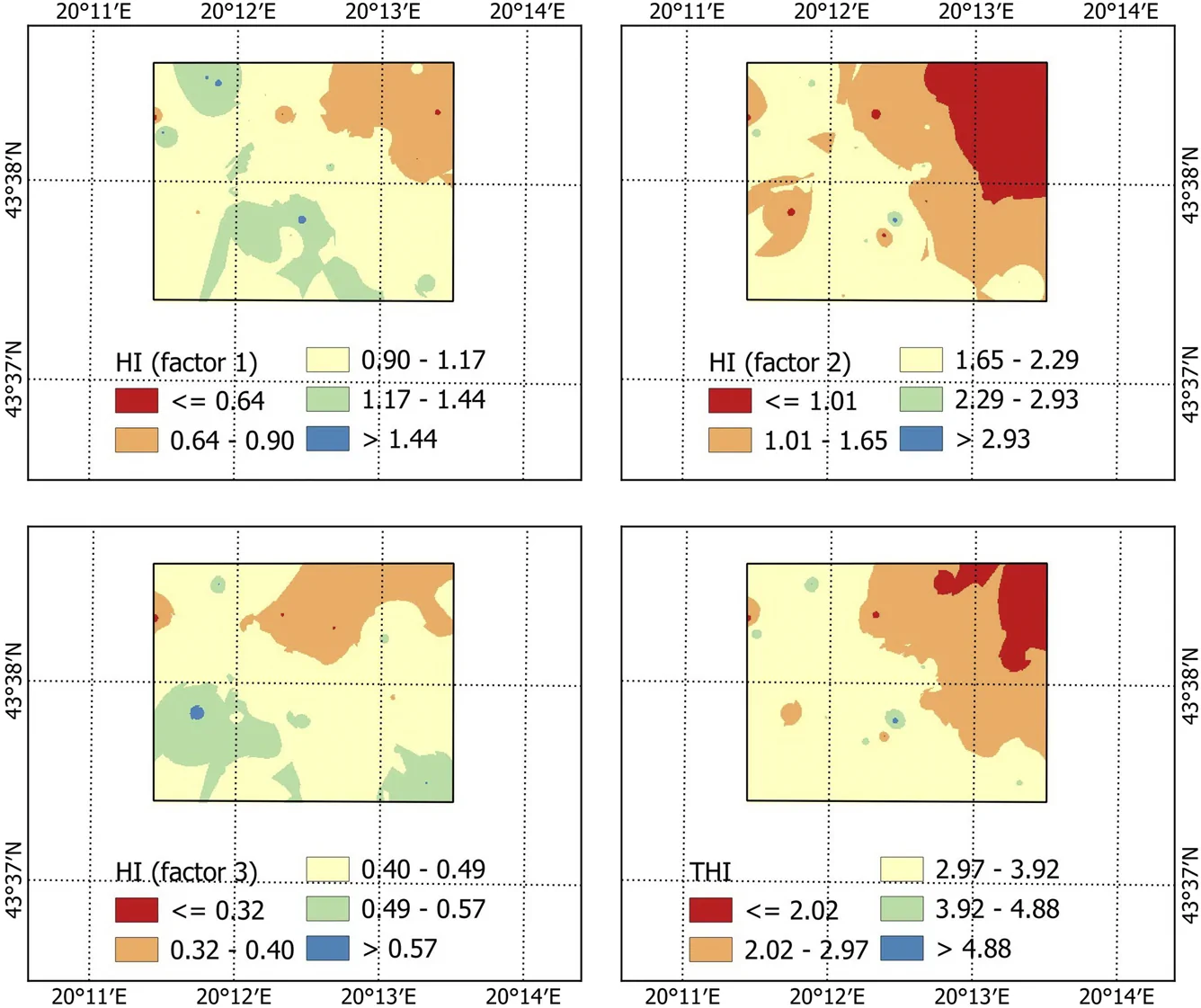
Spatial distribution of the contributions of individual positive matrix factorization (PMF) factors to the non-carcinogenic health risk in children. HI - hazard index for individual factors; THI - total hazard index.

In the case of TCR, Factors I and II display a nearly identical spatial distribution, indicating that As is the dominant element controlling the level of carcinogenic risk. Differences between Factors I and II are primarily in intensity, while the strength of Factor III is also notable (Figure 11; Supplementary Table S7). These results confirm the dominant influence of As a key element with significant effects on human health, consistent with previous studies, particularly in areas near antimony mines (Čakmak et al., 2025a). However, since the HRA was based on aqua regia-extractable concentrations, which do not necessarily reflect the bioaccessible fraction of PTEs, the estimated risks may overestimate actual human exposure.

**FIGURE 11:**
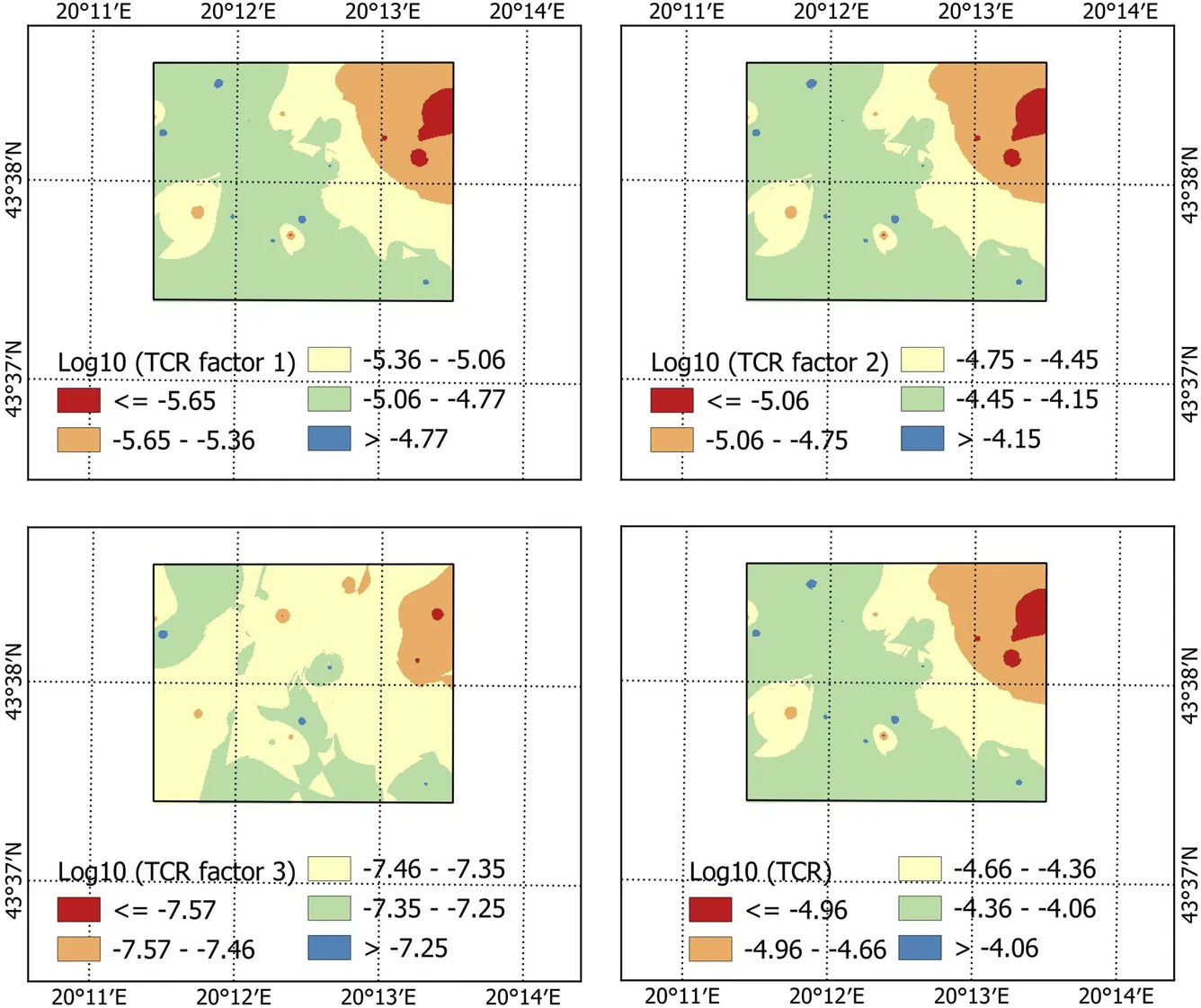
Spatial distribution of the contributions of individual positive matrix factorization (PMF) factors to total carcinogenic risk (TCR). TCR values are presented on a log_10_ scale.

## 4 Conclusion

Soil investigations near the former antimony mine by the village of Lisa, supported by the application of NA, CoDA, and PMF models, enabled an integrated assessment of PTE contamination and associated risks in the study area. Arsenic was identified as the dominant contaminant, with a mean concentration of 44.53 mg kg^-1^ and high variability among individual samples. The remediation value for As was exceeded in 37% of the samples, while 3.70% of the samples were classified as presenting a significant ecological risk. Although Cd exceeded the target value in 74.07% of the samples, the analysis of its variability and receptor model results indicated its predominantly geological origin. The combined application of NA, CoDA, and PMF confirmed the association of As and Sb with the anthropogenic factor, reflecting the long-term influence of former mining activities. The influence of this factor was spatially concentrated in the southwestern, central-western, and northeastern parts of the study area. Areas enriched in As were associated with elevated non-carcinogenic (HI = 2.89E+00) and carcinogenic risks (TCR = 6.10E-5), identifying priority areas for the implementation of appropriate management measures.

Based on the obtained results, former antimony mines may represent a long-term threat to ecosystem and human health, primarily due to associated PTEs, particularly As. Therefore, such mining areas require continuous monitoring of soil, water, and air, while agricultural crops should also be monitored to assess and prevent the transfer of PTEs into the food chain. Further investigations should include As speciation and its seasonal variability in relation to changes in soil moisture conditions. In areas with elevated As concentrations, appropriate remediation measures should also be considered, including the potential application of iron-modified biochar for As immobilization.

## Data Availability

The original contributions presented in the study are included in the article/Supplementary Material, further inquiries can be directed to the corresponding authors.
