# Supplementary material for: Multi-receptor models for source identification of potentially toxic elements near antimony mines and assessment of their health risks
Source: Front Toxicol. 2026 Oct 27;8:1878246. doi: 10.3389/ftox.2026.1878246 (PMC13749435; doi:10.3389/ftox.2026.1878246)
Supplement: Supplementary file 1 [file Supplementaryfile1.docx]

Supplementary Material

# 1 Supplementary Figures and Tables

## 1.1 Supplementary Tables

Table S1. Target (SW) and intervention (IW) values, and constants (A, B, C) for PTEs

Table S2. **Description and parameter values for health risk assessment of PTEs in soils**

Table S3. Relative bioavailability factor (RBA), oral reference dose (RfDo), dermal absorption fraction (ABSd), gastrointestinal absorption (GIABS), inhalation reference concentration (RfC), oral slope factor (CSFo) and inhalation unit risk (IUR) values

Table S4. Content of PTEs in individual samples

Table S5. Non-carcinogenic health risk for children

Table S6. Total carcinogenic risk

Table S7. Total non-carcinogenic health risk for children for individual samples in the identified factors

Table S8. Total carcinogenic health risk for individual samples in the identified factors

## 1.2 Supplementary Figures

Figure S1. Mean non-carcinogenic health risk values for children for individual PTEs by factors

Figure S2. Mean carcinogenic health risk values for individual PTEs by factors

Table S1. Target (SW) and intervention (IW) values, and constants (A, B, C) for PTEs

|  | SW | IW | A | B | C |
| --- | --- | --- | --- | --- | --- |
| mg kg^-1^ | | | / | | |
| As | 14.96* | 55.00 | 15.00 | 0.40 | 0.40 |
| Cd | 1.29* | 12 | 0.40 | 0.007 | 0.021 |
| Co | 18.34* | 240 | 2.00 | 0.28 | 0 |
| Cr | 96.73* | 380 | 50.00 | 2.00 | 0 |
| Cu | 31.30* | 190 | 15.00 | 0.60 | 0.60 |
| Ni | 80.85* | 210 | 10.00 | 1.00 | 0 |
| Pb | 63.88* | 530 | 50.00 | 1.00 | 1.00 |
| Sb | 3.93* | 15 | /, | / | / |
| Zn | 79.84* | 720 | 50.0 | 3.00 | 1.50 |

*Background value (MEP 2016)

Table S2. **Description and parameter values for health risk assessment of PTEs in soils**

| Symbol | Parameters (units) | | Values | | References |
| --- | --- | --- | --- | --- | --- |
| C | | PTE concentration (mg/kg) | | Site specific | |
| IRSres-c | Resident Soil Ingestion Rate - Child (mg day^-1^) | | 200 | | USEPA, 2020 |
| IRSres-a | Resident Soil Ingestion Rate - Adult (mg day^-1^) | | 100 | | USEPA, 2020 |
| RBA | relative bioavailability factor | | Arsenic=0.6 All Others=1 | | USEPA, 2020 |
| EFres | Resident Exposure Frequency Adult, Child (days year^-1^) | | 350 | | USEPA, 2020 |
| EDres-c | Resident Exposure Duration - child (years) | | 6 | | USEPA, 2020 |
| EDres-a | Resident Exposure Duration - adult (years) | | 20 | | EDres (26 years)-EDres-c (6 years) USEPA, 2020 |
| BWres-c | Resident Body Weight - child (kg) | | 15 | | USEPA, 2020 |
| BWres-a | Resident Body Weight - adult (kg) | | 80 | | USEPA, 2020 |
| ATres-c | Averaging time - resident child (days) | | 365 x EDres-c=2190 | | USEPA, 2020 |
| ATres-a | Averaging time - resident adult (days) | | 365 x EDres =7300 | | USEPA, 2020 |
| RfDo | Chronic Oral Reference Dose (mg kg^-1^-day) | | Contaminant specific | | Table S3 |
| SAres-c | Resident surface area soil - child (cm^2^ day^-1^) | | 2373 | | USEPA, 2020 |
| SAres-a | Resident surface area soil - adult (cm^2^ day^-1^) | | 6032 | | USEPA, 2020 |
| AFa | Skin adherence factor - adult (mg cm^-2^) | | 0,07 | | USEPA, 2020 |
| AFc | Skin adherence factor - child (mg cm^-2^) | | 0,2 | | USEPA, 2020 |
| ABSd | Fraction of contaminant absorbed dermally from soil (unitless) | | Contaminant specific | | Table S3 |
| GIABS | Fraction of contaminant absorbed in gastrointestinal tract (unitless) Note: if GIABS is >50% then it is set to 100% for calculation of dermal toxicity values | | Contaminant specific Inorganic default=1.0 VOC default=1.0 SVOC default=1.0 | | Table S3 |
| RfC | Chronic Inhalation Reference Concentration (mg m^-3^) | | Contaminant specific | | Table S3 |
| PEF | Particulate Emission Factor - Minneapolis (m^3^ kg^-1^) | | 1.36 x 109(region-specific) | | USEPA, 2020 |
| IFSres-adj | Resident Soil Ingestion Rate - age-adjusted (mg kg^-1^) | | Calculated using the age adjusted intake factors equation 36750 | | USEPA, 2020 |
| DFSres-adj | Resident soil dermal contact factor- age-adjusted (mg kg-^1^) | | Calculated using the age adjusted intake factors equation 103390 | | USEPA, 2020 |
| CSFo | | Oral Slope Factor (mg kg^-1^-day)^-1^ | | Table S3 | |
| IUR | Inhalation Unit Risk (μg m^-3^)^-1^ | | Contaminant specific | | Table S3 |
| LT | Life time (years) | | 76 | | Site specific |
| AT | Averaging time (days) | | 365*LT=27740 (Carcinogenic) | | Site specific |

Table S3. Relative bioavailability factor (RBA), oral reference dose (RfDo), dermal absorption fraction (ABSd), gastrointestinal absorption (GIABS), inhalation reference concentration (RfC), oral slope factor (CSFo) and inhalation unit risk (IUR) values

| PTE | RBA | RfDo | ABSd | GIABS | RfC | CSFo | IUR |
| --- | --- | --- | --- | --- | --- | --- | --- |
| As | 0.6 | 0.0003 | 0.03 | 1 | 0.000015 | 1.5 | 0.0043 |
| Cd | 1 | 0.003 | 0.001 | 0.25 | 0.0.00005 | / | 0.0018 |
| Co | 1 | 0.0003 | 0.001 | 1 | 0.000006 | / | 0.009 |
| Cr (III) | 1 | 1.5 | 0.001 | 1 | 0.000006 | / | / |
| Cu | 1 | 0.04 | 0.001 | 1 | 0.0024^b,c^ | / | / |
| Fe | 1 | 0.7 | 0.001 | 1 | / | / | / |
| Mn | 1 | 0.024 | 0.001 | 0.04 | 0.00005 | / | / |
| Ni | 1 | 0.02 | 0.001 | 0.04 | 0.000006 | / | 0.00026 |
| Pb | 1 | 0.0014^a^ | 0.001 | 0.15 | 0.0015^b,c^ | 0.0085^b,c^ | 0.000012^b,c^ |
| Sb | 1 | 0.0004 | 0.001 | 1 | 0.0003 | / | / |
| Zn | 1 | 0.3 | 0.001 | 1 | 0.035^b,c^ | / | / |
| Hg | 1 | 0.0003 | 0.001 | 0.007 | 0.3 | / | / |
| Source | | | | USEPA (2020) | | | |

^a^Jia et al., (2018); ^b^USDOE, (2011); ^c^Čakmak et al., (2020)

Table S4. Content of PTEs in individual samples

| Point | Geographic | |  |  |  |  |  |  |  |  |  |  |  |
| --- | --- | --- | --- | --- | --- | --- | --- | --- | --- | --- | --- | --- | --- |
| No. | Lati. | Long. | As | Cd | Co | Cr | Cu | Fe | Mn | Ni | Pb | Sb | Zn |
|  | x | y |  |  |  |  |  |  |  |  |  |  |  |
| 1 | 7437821 | 4834033 | 5.33 | 1.26 | 15.45 | 72.04 | 18.01 | 23730 | 643.67 | 33.86 | 20.90 | 2.47 | 63.10 |
| 2 | 7437477 | 4834417 | 12.83 | 1.11 | 12.78 | 48.99 | 12.15 | 19700 | 931.67 | 19.25 | 29.07 | 1.61 | 63.20 |
| 3 | 7435975 | 4833917 | 10.53 | 0.96 | 7.67 | 45.03 | 13.62 | 17900 | 411.72 | 27.15 | 16.25 | 1.38 | 44.76 |
| 4 | 7436709 | 4834260 | 7.97 | 0.96 | 9.99 | 40.10 | 16.07 | 19395 | 456.62 | 9.30 | 21.73 | 1.35 | 46.70 |
| 5 | 7435605 | 4833845 | 68.20 | 1.17 | 11.75 | 52.34 | 13.60 | 19175 | 729.67 | 24.74 | 26.20 | 5.08 | 60.40 |
| 6 | 7435786 | 4834127 | 46.88 | 1.48 | 15.60 | 60.54 | 14.08 | 24180 | 787.67 | 28.83 | 28.69 | 2.52 | 62.25 |
| 7 | 7435421 | 4832776 | 78.95 | 2.32 | 11.24 | 77.69 | 19.31 | 28530 | 805.17 | 39.24 | 27.15 | 3.67 | 79.35 |
| 8 | 7435033 | 4832827 | 7.25 | 2.60 | 16.29 | 127.79 | 44.33 | 42225 | 347.07 | 66.46 | 15.20 | 3.23 | 93.65 |
| 9 | 7437111 | 4833651 | 5.67 | 1.87 | 17.94 | 89.64 | 27.21 | 31500 | 625.67 | 62.51 | 21.69 | 2.48 | 85.70 |
| 10 | 7434547 | 4833878 | 10.75 | 0.80 | 3.92 | 37.25 | 7.99 | 16995 | 166.52 | 11.59 | 21.47 | 1.35 | 36.55 |
| 11 | 7436548 | 4833772 | 71.25 | 0.85 | 8.17 | 55.89 | 7.61 | 16800 | 148.62 | 16.25 | 25.29 | 2.56 | 31.55 |
| 12 | 7434643 | 4833713 | 107.45 | 2.63 | 9.41 | 90.54 | 18.67 | 27385 | 1043.67 | 40.07 | 52.50 | 2.59 | 75.35 |
| 13 | 7437706 | 4833941 | 4.08 | 1.20 | 8.73 | 72.14 | 15.17 | 22650 | 312.62 | 38.12 | 11.60 | 2.07 | 54.35 |
| 14 | 7437477 | 4833417 | 3.77 | 1.40 | 11.37 | 73.39 | 20.72 | 25440 | 302.97 | 43.85 | 13.57 | 2.20 | 59.45 |
| 15 | 7435134 | 4834324 | 48.62 | 1.38 | 18.69 | 57.79 | 13.88 | 20945 | 1320.17 | 23.11 | 32.45 | 2.41 | 65.35 |
| 16 | 7437203 | 4832995 | 18.65 | 0.95 | 15.62 | 48.88 | 9.31 | 17565 | 895.67 | 20.00 | 24.10 | 2.59 | 50.15 |
| 17 | 7435263 | 4834259 | 100.75 | 2.40 | 18.83 | 95.64 | 23.45 | 33200 | 725.17 | 39.88 | 31.55 | 3.45 | 76.30 |
| 18 | 7436505 | 4833343 | 111.60 | 2.47 | 13.00 | 93.39 | 17.57 | 29230 | 773.17 | 33.75 | 49.80 | 3.73 | 63.05 |
| 19 | 7435612 | 4831899 | 54.10 | 1.96 | 10.99 | 72.29 | 19.04 | 25145 | 643.17 | 31.93 | 36.78 | 3.65 | 77.20 |
| 20 | 7436493 | 4833392 | 21.37 | 0.72 | 11.72 | 41.45 | 6.41 | 14410 | 667.17 | 15.03 | 28.72 | 1.77 | 43.09 |
| 21 | 7437575 | 4832048 | 82.60 | 3.40 | 9.84 | 144.84 | 28.28 | 39705 | 582.17 | 48.66 | 39.55 | 4.01 | 97.00 |
| 22 | 7436537 | 4832950 | 21.04 | 1.45 | 14.15 | 84.59 | 19.02 | 24585 | 766.67 | 51.96 | 18.97 | 2.61 | 72.65 |
| 23 | 7437251 | 4832423 | 37.73 | 2.07 | 14.53 | 76.64 | 27.94 | 28500 | 680.67 | 59.26 | 23.30 | 2.26 | 87.95 |
| 24 | 7436069 | 4832570 | 4.92 | 1.38 | 13.45 | 84.69 | 18.81 | 24240 | 657.67 | 39.28 | 16.55 | 2.38 | 60.00 |
| 25 | 7437035 | 4832781 | 31.85 | 1.27 | 16.12 | 57.69 | 16.19 | 19850 | 778.67 | 25.55 | 23.81 | 1.99 | 51.85 |
| 26 | 7435869 | 4832510 | 92.25 | 2.78 | 10.65 | 91.74 | 25.67 | 33355 | 672.67 | 43.42 | 26.77 | 4.59 | 70.40 |
| 27 | 7436190 | 4832739 | 135.85 | 2.71 | 13.15 | 91.39 | 17.88 | 28055 | 818.67 | 36.40 | 47.77 | 8.60 | 56.65 |

Table S5. Non-carcinogenic health risk for children

| HI | As | Cd | Co | Cr | Cu | Fe | Mn | Ni | Pb | Sb | Zn | THI |
| --- | --- | --- | --- | --- | --- | --- | --- | --- | --- | --- | --- | --- |
| 1 | 1.53E-01 | 5.51E-03 | 6.62E-01 | 1.46E-03 | 5.78E-03 | 4.34E-01 | 3.72E-01 | 2.69E-02 | 7.65E-02 | 8.00E-02 | 2.70E-03 | 1.82E+00 |
| 2 | 3.68E-01 | 4.85E-03 | 5.47E-01 | 9.94E-04 | 3.90E-03 | 3.61E-01 | 5.39E-01 | 1.53E-02 | 1.06E-01 | 5.21E-02 | 2.70E-03 | 2.00E+00 |
| 3 | 3.02E-01 | 4.20E-03 | 3.29E-01 | 9.14E-04 | 4.37E-03 | 3.28E-01 | 2.38E-01 | 2.16E-02 | 5.95E-02 | 4.48E-02 | 1.91E-03 | 1.33E+00 |
| 4 | 2.28E-01 | 4.18E-03 | 4.28E-01 | 8.14E-04 | 5.15E-03 | 3.55E-01 | 2.64E-01 | 7.39E-03 | 7.96E-02 | 4.38E-02 | 2.00E-03 | 1.42E+00 |
| 5 | 1.95E+00 | 5.09E-03 | 5.03E-01 | 1.06E-03 | 4.36E-03 | 3.51E-01 | 4.22E-01 | 1.97E-02 | 9.59E-02 | 1.65E-01 | 2.58E-03 | 3.52E+00 |
| 6 | 1.34E+00 | 6.47E-03 | 6.68E-01 | 1.23E-03 | 4.52E-03 | 4.43E-01 | 4.56E-01 | 2.29E-02 | 1.05E-01 | 8.18E-02 | 2.66E-03 | 3.13E+00 |
| 7 | 2.26E+00 | 1.01E-02 | 4.81E-01 | 1.58E-03 | 6.19E-03 | 5.22E-01 | 4.66E-01 | 3.12E-02 | 9.94E-02 | 1.19E-01 | 3.39E-03 | 4.00E+00 |
| 8 | 2.08E-01 | 1.14E-02 | 6.98E-01 | 2.59E-03 | 1.42E-02 | 7.73E-01 | 2.01E-01 | 5.28E-02 | 5.56E-02 | 1.05E-01 | 4.00E-03 | 2.12E+00 |
| 9 | 1.62E-01 | 8.16E-03 | 7.68E-01 | 1.82E-03 | 8.72E-03 | 5.77E-01 | 3.62E-01 | 4.97E-02 | 7.94E-02 | 8.05E-02 | 3.66E-03 | 2.10E+00 |
| 10 | 3.08E-01 | 3.48E-03 | 1.68E-01 | 7.56E-04 | 2.56E-03 | 3.11E-01 | 9.63E-02 | 9.21E-03 | 7.86E-02 | 4.38E-02 | 1.56E-03 | 1.02E+00 |
| 11 | 2.04E+00 | 3.69E-03 | 3.50E-01 | 1.13E-03 | 2.44E-03 | 3.08E-01 | 8.60E-02 | 1.29E-02 | 9.26E-02 | 8.31E-02 | 1.35E-03 | 2.98E+00 |
| 12 | 3.08E+00 | 1.15E-02 | 4.03E-01 | 1.84E-03 | 5.99E-03 | 5.01E-01 | 6.04E-01 | 3.18E-02 | 1.92E-01 | 8.39E-02 | 3.22E-03 | 4.92E+00 |
| 13 | 1.17E-01 | 5.25E-03 | 3.74E-01 | 1.46E-03 | 4.86E-03 | 4.15E-01 | 1.81E-01 | 3.03E-02 | 4.25E-02 | 6.72E-02 | 2.32E-03 | 1.24E+00 |
| 14 | 1.08E-01 | 6.10E-03 | 4.87E-01 | 1.49E-03 | 6.64E-03 | 4.66E-01 | 1.75E-01 | 3.48E-02 | 4.97E-02 | 7.13E-02 | 2.54E-03 | 1.41E+00 |
| 15 | 1.39E+00 | 6.03E-03 | 8.01E-01 | 1.17E-03 | 4.45E-03 | 3.83E-01 | 7.64E-01 | 1.84E-02 | 1.19E-01 | 7.81E-02 | 2.79E-03 | 3.57E+00 |
| 16 | 5.34E-01 | 4.15E-03 | 6.69E-01 | 9.92E-04 | 2.98E-03 | 3.22E-01 | 5.18E-01 | 1.59E-02 | 8.82E-02 | 8.41E-02 | 2.14E-03 | 2.24E+00 |
| 17 | 2.89E+00 | 1.05E-02 | 8.07E-01 | 1.94E-03 | 7.52E-03 | 6.08E-01 | 4.19E-01 | 3.17E-02 | 1.16E-01 | 1.12E-01 | 3.26E-03 | 5.00E+00 |
| 18 | 3.20E+00 | 1.08E-02 | 5.57E-01 | 1.90E-03 | 5.63E-03 | 5.35E-01 | 4.47E-01 | 2.68E-02 | 1.82E-01 | 1.21E-01 | 2.69E-03 | 5.09E+00 |
| 19 | 1.55E+00 | 8.57E-03 | 4.71E-01 | 1.47E-03 | 6.10E-03 | 4.60E-01 | 3.72E-01 | 2.54E-02 | 1.35E-01 | 1.19E-01 | 3.30E-03 | 3.15E+00 |
| 20 | 6.12E-01 | 3.15E-03 | 5.02E-01 | 8.41E-04 | 2.06E-03 | 2.64E-01 | 3.86E-01 | 1.19E-02 | 1.05E-01 | 5.75E-02 | 1.84E-03 | 1.95E+00 |
| 21 | 2.37E+00 | 1.48E-02 | 4.21E-01 | 2.94E-03 | 9.07E-03 | 7.27E-01 | 3.37E-01 | 3.87E-02 | 1.45E-01 | 1.30E-01 | 4.15E-03 | 4.20E+00 |
| 22 | 6.03E-01 | 6.34E-03 | 6.06E-01 | 1.72E-03 | 6.10E-03 | 4.50E-01 | 4.43E-01 | 4.13E-02 | 6.95E-02 | 8.48E-02 | 3.11E-03 | 2.32E+00 |
| 23 | 1.08E+00 | 9.03E-03 | 6.22E-01 | 1.56E-03 | 8.96E-03 | 5.22E-01 | 3.94E-01 | 4.71E-02 | 8.53E-02 | 7.34E-02 | 3.76E-03 | 2.85E+00 |
| 24 | 1.41E-01 | 6.03E-03 | 5.76E-01 | 1.72E-03 | 6.03E-03 | 4.44E-01 | 3.80E-01 | 3.12E-02 | 6.06E-02 | 7.73E-02 | 2.56E-03 | 1.73E+00 |
| 25 | 9.12E-01 | 5.53E-03 | 6.90E-01 | 1.17E-03 | 5.19E-03 | 3.63E-01 | 4.50E-01 | 2.03E-02 | 8.72E-02 | 6.45E-02 | 2.22E-03 | 2.60E+00 |
| 26 | 2.64E+00 | 1.21E-02 | 4.56E-01 | 1.86E-03 | 8.23E-03 | 6.11E-01 | 3.89E-01 | 3.45E-02 | 9.80E-02 | 1.49E-01 | 3.01E-03 | 4.41E+00 |
| 27 | 3.89E+00 | 1.18E-02 | 5.63E-01 | 1.85E-03 | 5.73E-03 | 5.14E-01 | 4.74E-01 | 2.89E-02 | 1.75E-01 | 2.79E-01 | 2.42E-03 | 5.95E+00 |

Table S6. Total carcinogenic risk

| CR | As | Cd | Co | Ni | Pb | TCR |
| --- | --- | --- | --- | --- | --- | --- |
| 1 | 7.25E-06 | 5.47E-10 | 3.35E-08 | 2.12E-09 | 2.74E-07 | 7.56E-06 |
| 2 | 1.75E-05 | 4.82E-10 | 2.77E-08 | 1.21E-09 | 3.82E-07 | 1.79E-05 |
| 3 | 1.43E-05 | 4.17E-10 | 1.67E-08 | 1.70E-09 | 2.13E-07 | 1.46E-05 |
| 4 | 1.08E-05 | 4.15E-10 | 2.17E-08 | 5.83E-10 | 2.85E-07 | 1.12E-05 |
| 5 | 9.28E-05 | 5.06E-10 | 2.55E-08 | 1.55E-09 | 3.44E-07 | 9.32E-05 |
| 6 | 6.38E-05 | 6.43E-10 | 3.39E-08 | 1.81E-09 | 3.77E-07 | 6.42E-05 |
| 7 | 1.07E-04 | 1.01E-09 | 2.44E-08 | 2.46E-09 | 3.56E-07 | 1.08E-04 |
| 8 | 9.86E-06 | 1.13E-09 | 3.54E-08 | 4.17E-09 | 1.99E-07 | 1.01E-05 |
| 9 | 7.71E-06 | 8.10E-10 | 3.89E-08 | 3.92E-09 | 2.85E-07 | 8.04E-06 |
| 10 | 1.46E-05 | 3.45E-10 | 8.50E-09 | 7.27E-10 | 2.82E-07 | 1.49E-05 |
| 11 | 9.70E-05 | 3.67E-10 | 1.77E-08 | 1.02E-09 | 3.32E-07 | 9.73E-05 |
| 12 | 1.46E-04 | 1.14E-09 | 2.04E-08 | 2.51E-09 | 6.89E-07 | 1.47E-04 |
| 13 | 5.55E-06 | 5.21E-10 | 1.90E-08 | 2.39E-09 | 1.52E-07 | 5.73E-06 |
| 14 | 5.12E-06 | 6.06E-10 | 2.47E-08 | 2.75E-09 | 1.78E-07 | 5.33E-06 |
| 15 | 6.62E-05 | 5.99E-10 | 4.06E-08 | 1.45E-09 | 4.26E-07 | 6.66E-05 |
| 16 | 2.54E-05 | 4.12E-10 | 3.39E-08 | 1.25E-09 | 3.16E-07 | 2.57E-05 |
| 17 | 1.37E-04 | 1.04E-09 | 4.09E-08 | 2.50E-09 | 4.14E-07 | 1.38E-04 |
| 18 | 1.52E-04 | 1.07E-09 | 2.82E-08 | 2.12E-09 | 6.54E-07 | 1.53E-04 |
| 19 | 7.36E-05 | 8.51E-10 | 2.38E-08 | 2.00E-09 | 4.83E-07 | 7.41E-05 |
| 20 | 2.91E-05 | 3.13E-10 | 2.54E-08 | 9.43E-10 | 3.77E-07 | 2.95E-05 |
| 21 | 1.12E-04 | 1.47E-09 | 2.14E-08 | 3.05E-09 | 5.19E-07 | 1.13E-04 |
| 22 | 2.86E-05 | 6.30E-10 | 3.07E-08 | 3.26E-09 | 2.49E-07 | 2.89E-05 |
| 23 | 5.13E-05 | 8.97E-10 | 3.15E-08 | 3.72E-09 | 3.06E-07 | 5.17E-05 |
| 24 | 6.69E-06 | 5.99E-10 | 2.92E-08 | 2.46E-09 | 2.17E-07 | 6.94E-06 |
| 25 | 4.33E-05 | 5.49E-10 | 3.50E-08 | 1.60E-09 | 3.13E-07 | 4.37E-05 |
| 26 | 1.26E-04 | 1.20E-09 | 2.31E-08 | 2.72E-09 | 3.51E-07 | 1.26E-04 |
| 27 | 1.85E-04 | 1.17E-09 | 2.85E-08 | 2.28E-09 | 6.27E-07 | 1.86E-04 |

Table S7. Total non-carcinogenic health risk for children for individual samples in the identified factors

| No. | THI  Factor I | THI  Factor II | THI  Factor III |
| --- | --- | --- | --- |
| 1 | 8.73E-01 | 4.89E-01 | 4.51E-01 |
| 2 | 9.65E-01 | 6.62E-01 | 3.68E-01 |
| 3 | 5.57E-01 | 4.78E-01 | 2.93E-01 |
| 4 | 6.37E-01 | 4.52E-01 | 3.24E-01 |
| 5 | 1.18E+00 | 1.95E+00 | 3.94E-01 |
| 6 | 1.19E+00 | 1.48E+00 | 4.57E-01 |
| 7 | 1.29E+00 | 2.23E+00 | 4.73E-01 |
| 8 | 8.50E-01 | 5.95E-01 | 6.68E-01 |
| 9 | 9.67E-01 | 5.54E-01 | 5.71E-01 |
| 10 | 3.57E-01 | 4.26E-01 | 2.35E-01 |
| 11 | 8.06E-01 | 1.87E+00 | 2.97E-01 |
| 12 | 1.54E+00 | 2.93E+00 | 4.39E-01 |
| 13 | 5.22E-01 | 3.49E-01 | 3.62E-01 |
| 14 | 6.00E-01 | 3.77E-01 | 4.24E-01 |
| 15 | 1.50E+00 | 1.60E+00 | 4.57E-01 |
| 16 | 1.04E+00 | 8.06E-01 | 3.87E-01 |
| 17 | 1.59E+00 | 2.81E+00 | 5.99E-01 |
| 18 | 1.54E+00 | 3.04E+00 | 5.03E-01 |
| 19 | 1.07E+00 | 1.63E+00 | 4.36E-01 |
| 20 | 8.44E-01 | 7.94E-01 | 3.04E-01 |
| 21 | 1.25E+00 | 2.36E+00 | 5.81E-01 |
| 22 | 9.85E-01 | 8.66E-01 | 4.57E-01 |
| 23 | 1.07E+00 | 1.27E+00 | 5.02E-01 |
| 24 | 8.20E-01 | 4.64E-01 | 4.35E-01 |
| 25 | 1.08E+00 | 1.10E+00 | 4.12E-01 |
| 26 | 1.31E+00 | 2.56E+00 | 5.27E-01 |
| 27 | 1.73E+00 | 3.65E+00 | 5.50E-01 |
| Average | 1.04E+00 | 1.40E+00 | 4.41E-01 |
| MAX | 1.73E+00 | 3.65E+00 | 6.68E-01 |
| Unacceptible  Risk  % | 44.44 | 44.44 | 0.00 |

Table S8. Total carcinogenic health risk for individual samples in the identified factors

| No. | TCR  Factor I | TCR  Factor II | TCR  Factor III |
| --- | --- | --- | --- |
| 1 | 1.55E-06 | 5.98E-06 | 3.47E-08 |
| 2 | 3.56E-06 | 1.43E-05 | 4.25E-08 |
| 3 | 2.86E-06 | 1.17E-05 | 2.51E-08 |
| 4 | 2.24E-06 | 8.89E-06 | 3.17E-08 |
| 5 | 1.80E-05 | 7.52E-05 | 4.27E-08 |
| 6 | 1.24E-05 | 5.17E-05 | 4.64E-08 |
| 7 | 2.08E-05 | 8.70E-05 | 4.51E-08 |
| 8 | 2.01E-06 | 8.06E-06 | 3.03E-08 |
| 9 | 1.65E-06 | 6.35E-06 | 3.83E-08 |
| 10 | 2.95E-06 | 1.19E-05 | 2.84E-08 |
| 11 | 1.88E-05 | 7.85E-05 | 3.94E-08 |
| 12 | 2.84E-05 | 1.18E-04 | 7.55E-08 |
| 13 | 1.15E-06 | 4.55E-06 | 2.03E-08 |
| 14 | 1.09E-06 | 4.22E-06 | 2.43E-08 |
| 15 | 1.29E-05 | 5.37E-05 | 5.23E-08 |
| 16 | 5.05E-06 | 2.06E-05 | 3.87E-08 |
| 17 | 2.65E-05 | 1.11E-04 | 5.58E-08 |
| 18 | 2.95E-05 | 1.23E-04 | 7.43E-08 |
| 19 | 1.44E-05 | 5.97E-05 | 5.40E-08 |
| 20 | 5.78E-06 | 2.37E-05 | 4.19E-08 |
| 21 | 2.18E-05 | 9.11E-05 | 5.95E-08 |
| 22 | 5.63E-06 | 2.33E-05 | 3.36E-08 |
| 23 | 1.00E-05 | 4.16E-05 | 4.04E-08 |
| 24 | 1.41E-06 | 5.50E-06 | 2.88E-08 |
| 25 | 8.49E-06 | 3.52E-05 | 3.99E-08 |
| 26 | 2.43E-05 | 1.02E-04 | 4.55E-08 |
| 27 | 3.58E-05 | 1.50E-04 | 7.38E-08 |
| Average | 1.18E-05 | 4.91E-05 | 4.31E-08 |
| MAX | 3.58E-05 | 1.50E-04 | 7.55E-08 |
| Notable  risk  % | 48.15 | 74.07 | 0.00 |

Figure S1. Mean non-carcinogenic health risk values for children for individual PTEs by factors

Figure S2. Mean carcinogenic health risk values for individual PTEs by factors

**References:**

Čakmak, D., Perović, V., Kresović, M., Pavlović, D., Pavlović, M., Mitrović, M., Pavlović, P. (2020). Sources and a health risk assessment of potentially toxic elements in dust at children’s playgrounds with artificial surfaces: a case study in Belgrade. Arch. Environ. Contam. Toxicol. 78, 190–205. <https://doi.org/10.1007/s00244-019-00702-0>

Jia, Z., Li, S., Wang, L. (2018). Assessment of soil heavy metals for eco-environment and human health in a rapidly urbanization area of the upper Yangtze Basin. Sci. Rep. 8, 3256. <https://doi.org/10.1038/s41598-018-21569-6>

MEP (2016). The state of non-agricultural land in industrial zones of larger cities in the Republic of Serbia from the aspect of biological and chemical quality (in Serbian). Available at: <https://www.ekologija.gov.rs/sites/default/files/old-documents/Zemljiste/Projekti/Stanje-zemljista-industrijskih-zona-vecih-gradova4.pdf> (Accessed October 2, 2023).

USDOE (2011). The risk assessment information system (RAIS). U.S. Department of Energy, Oak Ridge Operations Office.

USEPA (2020). Regional screening levels (RSLs) user’s guide. Available at: <https://www.epa.gov/risk/regional-screening-levels-rsls-users-guide> (Accessed October 2, 2023).
